# Supplementary material for: Sustainable poverty alleviation capacity construction of farmers in poverty-stricken areas under the background of rural revitalization
Source: PLoS One. 2022 Dec 15;17(12):e0276804. doi: 10.1371/journal.pone.0276804 (PMC9754586; doi:10.1371/journal.pone.0276804)
Supplement: S1 File — This is a sample of the questionnaire. (DOC) [file pone.0276804.s001.doc]

Name of Village： County Town Village No.

Questionnaire No.: County No.: Village No.: Sample No.:

**Questionnaire on Farmers and Poverty Alleviation**

**in Poor Areas of Yunnan Province**

Dear Sir / Madam

Thank you for taking the time to participate in our survey. This survey is anonymous, and the answers do not involve right or wrong. Please answer according to the actual situation. Your answer will help us to further improve the poverty alleviation and poverty reduction work in Yunnan's poor areas. Thank you for your support and cooperation!

Research Group

February 2019

**I, Family Population Characteristics**

1. There are members in your family who live together or have close relationships. Among them, there are males, among the males, there are members who are capable of working; There are females, among the females, there are members who are capable of working. family members aged 18 to 60.

2. The head of your household is male / female, aged , with/without working experience, and the education level is:

A. Illiteracy B. Primary school C. Junior high school D. Senior high school or technical secondary school E. College or vocational college F. Bachelor's degree or above

3. In your family, there are family members who are studying or graduated with college degree or above, and there are family members who have junior high school education or above.

4. In the past 5 years, whether or not your family members have suffered from serious diseases, whether or not serious disabilities; In the past 2 years, your family's medical expenses are:

A. Less than 3,000 yuan B. 3,000 to 10,000 yuan C. 10,000 to 20,000 yuan

D. 20,000 yuan or more

5. In your family, there are ethnic minorities, and members believe in religion and participate in religious organizations or activities.

6. village cadres in your family.

**II, Overview of Family Life**

1. Your family's living years in the local place are:

A. Less than one year B. More than one year but less than three years C. More than three years but less than five years D. More than five years

2. The land allocated to your family totals mu, and the land actually cultivated by your family members is mu. The terrain of the land is:

A. Flat land B. Mountain land C. Terrace D. Paddy field F. Others

3. The residential area of your family is square meters, and the type is:

A. Brick concrete building B. Brick concrete bungalow C. Adobe bungalow

D. Others

4. If a similar house is to be built now, it will probably cost yuan.

5. Residential supporting facilities include (multiple choices):

A. Well or tap water B. Independent toilet C.Lighting or cooking electricity

D. Biogas E. Others

6. Household appliances owned by the family include (multiple choices):

A. TV B. Washing machine C. Refrigerator D. Recorder or DVD player

E. Radio F. Computer G. Mobile phone H. Others

hours you watch TV every day

7. Do you know about your neighbors:

A. Very familiar B. Quite familiar C. Average D. Not very familiar E. Not at all

8. Whether the transportation around your family is convenient:

A. Very convenient B. Relatively convenient C. Ordinary

D. Relatively inconvenient E.Very inconvenient

9. Do you think the work of the village cadres needs to be improved:

A. Very necessary B. Necessary C. Keep the present situation

D. Not necessary E. Not necessary at all, it's very good now.

**III, Overview of Family Economic Activities**

1. 5 years ago (in 2012), your family's annual total income was about yuan, yuan last year (in 2017), your family's income level has / has not improved significantly, increasing by about percent.

2. Compared with 5 years ago (in 2012), last year (in 2017) your family's overall living conditions have / have not improved significantly:

A. A lot of improvement B. A little Improvement C. No improvement or no comments

D. Decline in living conditions E. Decline a lot

3. In the past 3 years, your family's stored grain is generally available for months, with / without purchased grain or receiving or handouts from others.

4. In the past 5 years, your family's income from the planting industry accounts for about the total income:

A. 20% to 40% B. 40% to 60% C. 60% to 80% D. More than 80%

5. The impact of agricultural tax exemption on your family's production:

A. Very helpful B. Relatively helpful C. Not very helpful D. Negative impact

6. In addition to planting, your family also engages in:

A. Breeding B. Food processing and operation C. Wholesale and retail

D. Housing construction E. Agricultural tools manufacturing F. Agricultural leisure

G. working in other place H. Others (please specify)

7. Your family has / does not have agricultural vehicles, has / does not have tractors, has / does not have other agricultural machinery, and has / does not have workers who can drive cars or tractors.

8. Which of the following production measures have been taken in your farmland:

A. Greenhouse B. Irrigation machinery C. Regular application of pesticides and fertilizers D. Others

9. The main marketing channels of your family's agricultural products are:

A. Local sale B. Door-to-door purchase C. Enterprise purchase D. County sale

E. Purchase station F. Others

10. The channels for you to obtain agricultural product sales information are:

A. Other farmers B. Village cadres C. TV D. Network E. Radio F. Others

11. Your family has / has not participated in production mutual aid organizations, has /has not had long-term cooperative relations with agricultural enterprises, and these enterprises or organizations have / have not obviously helped your family's production:

A. Very helpful B. Average C. No help D. Negative influence

E. Great negative influence

12. In the past 3 years, your family members have / have not participated in production skills training. The initiators of such training are:

A. Agricultural technology promotion station B. Enterprise

C. Superior government D. Other organizations

13. This kind of training is / is not helpful to your family's productive capacity:

A. Great help B. certain help C. No help D. Negative influence

E. Great negative influence

12. In the past 3 years, of your family members has worked in other place for more than six months every year. Whether or not they have brought great help to the family, mainly reflected in:

A. Capital B. New market information C. New production technology

D. New life concept

13. Their main destinations for working are:

A. Neighboring Village B. County C. Provincial Capital D. Other Province

14. The top three items of your family's expenditure are (multiple choices):

A. Purchase of food B. purchase of means of agricultural production C. Purchase of daily necessities D. Education and medical treatment E.Weddings and funerals

F. Purchase of agricultural machinery G. Purchase of household appliances

H. Going out I. Others

**IV, Participation in Poverty Alleviation**

1. In the past 3 years, whether or not your region has encountered serious natural disasters are (multiple choice):

A. Drought B. Flood C. Geological disaster D. Animal and plant diseases and pests

2. To increase your income for yourself and your family:

A. Very difficult B. Difficult C. Can be achieved. D. Achieved E. Easy to achieve

3. If the income level remains unchanged, it will affect you and your family;

A. Extremely B. Very C. Ordinary D. A little E. No impact

4. Whether or not your family has participated in the New Rural Cooperative Medical Insurance, whether or not received the medical assistance in the past 3 years, whether or not helps your family.

5. Whether or not your family has participated in poverty assistance projects (such as Subsistence Allowances) for years. The sponsors of these projects are:

A. Government B. Enterprise C. Individual D. Non-governmental organization

6. Whether or not these projects have significantly improved your family's living standard:

A. Very helpful B. Average C. No help D. Negative influence

E. Great negative influence

Mainly in (multiple choices):

A. Give necessities and funds B. Bring new technologies and new varieties

C. Give start-up funds for production development D. Bring new sales channels

E. Improve production facilities and living environment F. Others

7. If you do not participate in any assistance projects, the specific reasons are (multiple choices):

A. I don't know these projects B. I know but don't know how to apply for these projects C. These projects are not helpful D. I have applied for these projects but haven't been approved E. No one has helped me to obtain these projects

F. No poverty alleviation projects

8. Whether or not your family needs to take the initiative to obtain funding for these projects, whether or not express gratitude to the relevant village cadres, and whether or not you think the project implementation process is fair:

A. Very good B. Good C. Average D. Poor E. Very poor

9. Which aspect of poverty alleviation work do you think will help your family most (multiple choices):

A. Road facilities B. Power and water supply C. Education and training

D. Housing construction E. Income subsidy F. Employment support

G. Medical assistance H. Production cooperation I. Market development

J. Others

10. The channels for your family to obtain funds are:

A. Credit Cooperatives B. Small Bank loans C. Loans to the enterprises

D. Loans to familiar people

11. Your family receives funds:

A. Extremely difficult B. Very difficult C. Difficult D. Not difficult

12. Whether or not your family has received cash assistance, such opportunities:

A. Few B. Less C. Average D. More E. A lot

13. Whether or not your family participated in the village activities last year, which mainly provide:

A. Small and micro finance B. Health care C. Technical training D. Facility maintenance and construction E. Resource development F. Small and micro enterprises

14. If there are poverty alleviation projects in the village that need to work for free, whether or not your family willing to work.

15. As you know, whether or not the government, enterprises or organizations have formulated different support measures according to the needs of different farmers.

16. Among the assistance projects you have participated in, the ones you have most contact with are:

A. County government staff B. Township government staff C. Village cadres

D. Volunteers

17. If your family encounters major difficulties, who do you tend to for help:

A. County government B. Township government C. Village cadres

D. Volunteers E. Neighbors F. Relatives

18. What are your further expectations or requirements for the assistance project:

This is the end of the questionnaire. Thank you for your answer!
