# Supplementary material for: Sustainable poverty alleviation capacity construction of farmers in poverty-stricken areas under the background of rural revitalization
Source: PLoS One. 2022 Dec 15;17(12):e0276804. doi: 10.1371/journal.pone.0276804 (PMC9754586; doi:10.1371/journal.pone.0276804)
Supplement: Attachment — Submitted filename: Response to Editors-1011.docx [file pone.0276804.s003.docx]

Revision details of the manuscript “PONE-D-22-10030_R1” following the suggestions of editors

**Title of paper:** Sustainable poverty alleviation capacity construction of farmers in poverty-stricken areas under the background of rural revitalization

Dear editors

Thank you for your valuable suggestions concerning our manuscript, which have been very helpful for revising and improving our paper to enhance the impact of our research. Our response to each of the suggestions is provided below, and changes in the revised version of the manuscript.

1. **We looked up the instructions for “Our new trial integration gives PLOS Pathogens authors the option to upload data files directly to Dryad Digital Repository from the manuscript submission system.”** **But we did not find the corresponding upload path in the system.**

**We updated the data availability statement in manuscript 305-309 and uploaded the sample survey questionnaire and relevant statistics into the author's system.**

2. **We choose to disclose the questionnaire samples and statistical data of the manuscript, and Anyi Xing is responsible for this part. We added an author who participated in the questionnaire survey and data collection related to the manuscript, which we explained in line 296-297 of the manuscript.**
